# Statistical Workflow for Feature Selection in Human Metabolomics Data

**DOI:** 10.3390/metabo9070143

**Published:** 2019-07-12

**Authors:** Joseph Antonelli, Brian L. Claggett, Mir Henglin, Andy Kim, Gavin Ovsak, Nicole Kim, Katherine Deng, Kevin Rao, Octavia Tyagi, Jeramie D. Watrous, Kim A. Lagerborg, Pavel V. Hushcha, Olga V. Demler, Samia Mora, Teemu J. Niiranen, Alexandre C. Pereira, Mohit Jain, Susan Cheng

**Affiliations:** 1 Department of Statistics, University of Florida, Gainesville, FL 32611, USA; 2 Cardiovascular Division, Brigham and Women’s Hospital, Harvard Medical School, Boston, MA 02115, USA; 3 Smidt Heart Institute, Cedars-Sinai Medical Center, Los Angeles, CA 90048, USA; 4 Departments of Medicine & Pharmacology, University of California San Diego, La Jolla, CA 92093, USA; 5 Preventive Medicine, Brigham and Women’s Hospital, Harvard Medical School, Boston, MA 02115, USA; 6 National Institute for Health and Welfare, FI 00271 Helsinki, Finland; 7 Department of Medicine, Turku University Hospital and Univesity of Turku, FI 20521 Turrku, Finland; 8 Department of Genetics, Harvard Medical School, Boston, MA 02115, USA; 9 Framingham Heart Study, Framingham, MA 01701, USA

**Keywords:** statistical methods, large-scale metabolomics, high-dimensional data

## Abstract

High-throughput metabolomics investigations, when conducted in large human cohorts, represent a potentially powerful tool for elucidating the biochemical diversity underlying human health and disease. Large-scale metabolomics data sources, generated using either targeted or nontargeted platforms, are becoming more common. Appropriate statistical analysis of these complex high-dimensional data will be critical for extracting meaningful results from such large-scale human metabolomics studies. Therefore, we consider the statistical analytical approaches that have been employed in prior human metabolomics studies. Based on the lessons learned and collective experience to date in the field, we offer a step-by-step framework for pursuing statistical analyses of cohort-based human metabolomics data, with a focus on feature selection. We discuss the range of options and approaches that may be employed at each stage of data management, analysis, and interpretation and offer guidance on the analytical decisions that need to be considered over the course of implementing a data analysis workflow. Certain pervasive analytical challenges facing the field warrant ongoing focused research. Addressing these challenges, particularly those related to analyzing human metabolomics data, will allow for more standardization of as well as advances in how research in the field is practiced. In turn, such major analytical advances will lead to substantial improvements in the overall contributions of human metabolomics investigations.

## 1. Introduction

Rapid advances in mass spectrometry (MS) technologies have enabled the generation of large-scale metabolomics data in human studies. These technical advances have outpaced the development of statistical methods for handling and analyzing datasets now of burgeoning size and complexity [1,2,3]. Early investigations using metabolomics technologies have applied a variety of statistical methods in analyses of datasets containing up to 200 metabolite measures, typically acquired from a targeted metabolomics platform collected from human studies involving tens to hundreds of observations [4,5]. Nontargeted metabolomics platforms now allow the measurement of up to tens of thousands of metabolites, most which are unknown molecular species that demonstrate varying levels of intercorrelations within a given dataset as well as correlations with a given clinical outcome [6]. Furthermore, current metabolomics technologies have augmented throughput capacity that can facilitate data collection for thousands of observations per human cohort experiment [7,8]. At present, however, there are no existing standard protocols for analyzing these increasingly complex metabolomics data. Thus, herein, we review the available statistical methods for analyzing high-dimensional metabolomics data in the setting of a clinical study. We also outline an accessible yet flexible approach that can be used to optimize sensitivity and specificity for identifying potentially important metabolites associated with a clinically relevant outcome.

## 2. Analytical Challenges

Due to the increasing complexity of metabolomics data, combined with the variety of different study designs employed in practice, customized approaches are often required for analyzing metabolite variation in relation to clinical outcomes. Notwithstanding differences in study design and data structure, common to almost all metabolomics datasets is the need to address certain statistical considerations (Table 1). An initial key consideration is missingness, given that all metabolomic data invariably demonstrate patterns of missing values that are often but not always more frequent for low abundant metabolites. Contributing factors include underlying biology (e.g., very low or true zero values due to biological differences between healthy and disease-enriched cohorts) and technical issues (e.g., actual values below the detection limits for a given method, which may or may not be rectified using different or complementary methods). The etiology of missing values will vary, at least in part, based on the platform used to profile metabolites (i.e., nuclear magnetic resonance [NMR] spectroscopy, gas chromatography mass spectrometry [GC-MS], liquid chromatography mass spectrometry [LC-MS]). In rare circumstances, for certain types of metabolites (e.g., derivatives of known toxic exposures), missing values may be most appropriately coded as true zero values as opposed to imputed. There is some research on the best methods for dealing with missing data in the context of metabolomics [9,10,11]. The appropriate choice depends on whether the authors believe their data is missing at random (MAR) or missing not at random (MNAR) [12]. Data that are MAR are missing due to observed factors that can be modeled, while MNAR data would occur if the data are missing due to unobserved factors. In metabolomics, MNAR can occur when data are missing because they are below the limit of detection. If it is believed that data are missing due to limit of detection issues then users can impute the missing values using quantile regression imputation of censored data (QRILC), which is available in the R package imputeLCMD [13]. This was shown to outperform the standard approach of simply filling in missing values with half the minimum observed value [9]. If users believe the data are MAR, then they should be imputed using multiple imputation strategies. Recent studies have seen that both random forest or k-nearest neighbors imputation work well [9,10]. Regardless of the imputation strategy used, we recommend users impute the missing values as failing to do so can lead to underestimation of standard errors and potentially biased estimates of effect of interest.

For non-zero metabolite variables, transformation is usually recommended due to frequently right-skewed distributions [14]. Bimodal or multimodal distributions may also be seen, albeit less commonly. While many statistical approaches do not rely on the distribution of metabolites being symmetric, transformation can help reduce the impact of outliers when the distribution is highly skewed. Irrespective of distribution patterns, metabolite variables almost always demonstrate intercorrelations. The extent to which intercorrelations exist between metabolites will vary between datasets due to a variety of factors, including those related to study sampling as well as technical issues. Dealing with correlations between metabolites depends on the scientific goal of the study. If the goal is to predict the outcome given the metabolites, or to understand if any of the metabolites are predictive of the outcome, then dimension reduction tools such as principal components analysis (PCA) [15] can be used to produce a set of independent predictors from the original, highly correlated metabolites. If, however, the interest is in the identification of important metabolites, then these tools can not be applied and the original, correlated metabolites must be used. If the variables are extremely correlated then identifying individual metabolites might be difficult without a huge sample size, but groups of potentially important metabolites can be identified using the tools we discuss in the following section.

Within a given study sample, metabolite variation will be influenced by time-dependent factors given that a portion of the human metabolome changes dynamically in response to acute perturbation or stress while another portion of the metabolome exhibits relatively little change over time, except in response to major chronic exposures. Further, the effects of metabolites on an outcome could be influenced by confounding factors such as demographic variables or environmental exposures. To account for this, the confounding factors must be adjusted for if the interest is in the effects of particular exposures on an outcome. One such way to do this is to use a regression model that adjusts for both the metabolite values and the confounding factors so that the metabolite effect is the effect after conditioning on levels of confounding factors. Many of the approaches in the following section can be adopted to adjust for confounding variables and should be done if the interest is in the effect of a metabolite on an outcome.

## 3. Statistical Methods for Analyzing Metabolomics Data

A variety of different statistical methods are available for analyzing high-dimensional metabolomics data. Many of these approaches are the same as those used with other omics based data sources such as genomics, and a number of studies have looked to evaluate the performance of some of these approaches in the context of high-dimensional omics data [16,17,18,19]. Methods that have been applied in prior or ongoing clinical metabolomics studies are summarized in Table 2 and are also described in more detail herein. We want to stress that the choice of statistical approach depends heavily on the research question. Throughout, we will both explain the use of each method, as well as discuss the types of research questions they can be used for.

Univariate analyses with multiple testing correction, such as the Bonferroni correction for controlling the global type I error rate or the Benjamini–Hochberg correction for controlling the false discovery rate (FDR) [1], have previously been applied in a variety of predominantly metabolomics targeted studies [2,3,20]. This approach involves M tests, where M is the total number of metabolite variables analyzed separately in relation to an outcome of interest. The P value for each separate metabolite test can be considered significant or non-significant based on a P value threshold that is corrected to account for the fact that multiple hypotheses are being tested. The Bonferroni method is commonly used and deems a metabolite significant if it is smaller than 0.05 / M, while other corrections can be used to control the false discovery rate. Overall, univariate analysis with multiple testing correction is an attractive approach because it is simple to implement and provides a measure of statistical significance for each covariate that is easy to interpret. Further, it is very easy to adjust for confounding factors when running univariate analyses as they can be included in a regression model as additional predictors. However, this approach alone does not account for associations between metabolites and outcomes that are conditional on other metabolite values. For instance, a given metabolite may appear significantly associated with an outcome in isolation but does not demonstrate a significant association when other metabolite associations are taken into account. Furthermore, approaches to account for multiple testing, such as the Bonferroni correction or even FDR, can be very conservative in the setting of a large number of analyzed metabolites, leading to limited statistical power overall.

The principle components analysis (PCA) approach [15] is designed to reduce the dimension of the number of metabolites being analyzed, assuming there is substantial correlation between metabolites in a given dataset. Thus, PCA directly addresses the issue of intercorrelatedness and has been used in prior human metabolomics studies, usually in combination with other methods [21,22,23,24,25,26]. The PCA approach takes the original metabolites and finds linear combinations of these that are orthogonal to each other and that explain the most variation in the metabolite dataset. The PCA approach can be used to identify metabolite combinations associated with a given outcome, but PCA is not intrinsically designed to identify original predictor variables (i.e., metabolites) of importance. A varimax rotation can be used to increase the interpretability of principal components, given that principal components are comprised of a small subset of the original metabolites [27], but this may not improve things greatly with large numbers of metabolites. Those metabolites contributing to a given principal component can be ranked by the importance of their contributions. However, for each contributing metabolite, a measure of the magnitude of its association with given outcome is not provided, and a test of the significance of association is also not provided. PCA can be used to show separation in two groups by visually plotting their first and second principle components against each other [28], or can be used in prediction modeling by reducing the dimension of the predictors [29]. Generally though, PCA should not be used for identifying individual important predictors.

The partial least squares (PLS) regression method [30] aims to maximize the covariance between a matrix of metabolites and a continuous outcome (or categorical outcome using the PLS discriminant analysis (DA) variation [31]) by decomposing metabolite and outcomes data into latent structures. Cross validation can be used to select the optimal number of latent variables that are used. This approach aims to maximize the covariance between the outcome and matrix of metabolites by projecting both to linear subspaces of the original variables. There has been widespread use of PLS in metabolomics studies [21,22,23,24,32,33,34,35], although it comes with some limitations with regard to metabolite selection. While finding reduced dimensions that can explain the outcome variable, PLS regression generally provides only a measure of variable importance and does not naturally perform variable selection, although a number of ad hoc approaches for variable selection have been proposed [36,37]. Nonetheless, no clear-cut approach is best for determining which metabolites are actually important in predicting the outcome. Sparse extensions of both approaches have been proposed [34,38,39,40,41], which add a penalty to the loading scores forcing some of the variables to have zero weight in the final model. One can take the list of non-zero metabolites to be the metabolites that are deemed important. This is potentially a very fruitful direction for identifying important metabolites that jointly predict the outcome well. The resulting set of variables can be substantially different from the variables selected by univariate methods that analyze each metabolite separately. To this end, multivariate PLS methods can be implemented using the SPLS package in R [39].

The linear discriminant analysis (LDA) approach has also been used in metabolomic studies and aims to find linear combinations of metabolite variables that are best able to separate classes of a categorical outcome [25,26]. Although LDA cannot be used for a continuous outcome, such an outcome may be discretized for the application of LDA. Conventionally, LDA will perform poorly or can even fail completely when the number of covariates exceeds the number of subjects [42,43], a common feature of metabolomics datasets. One could use principle components first and then run discriminant analysis; however, this approach will suffer from the same interpretational problems as PCA by not allowing for identification of important metabolites. Furthermore, the LDA approach does not intrinsically identify a set of important variables and can only be used to create variable rankings or importance measures. For this reason, we consider a sparse version of LDA [44,45,46], which again adds a penalty for the variable loadings and, thus, allows for simultaneous variable selection. As with PCA and PLS, LDA can provide a measure of variable importance that can be used for metabolite ranking.

The least absolute shrinkage and selection operator (LASSO) approach aims to fit a model that regresses the outcome against all of the metabolites simultaneously and applies a penalty to the magnitude of regression coefficients to achieve sparse variable selection [47]. Traditional regression models are not infeasible when the number of metabolites is larger than the sample size [48], since the model becomes overfit and the parameter estimates become meaningless. To address this issue, the LASSO applies a penalty to the magnitude of the regression coefficients, which forces the regression coefficients for many coefficients to be zero while also shrinking others in magnitude. LASSO has been shown to be useful in both prediction and metabolite selection [49,50]. One can take the collection of metabolites with non-zero regression coefficients from LASSO to be “significant” in the sense that they are associated with the outcome. This should not be confused with statistical significance in the general sense of P values and rejecting null hypotheses; however, it is a powerful tool for variable selection. To perform LASSO, one must also select a tuning parameter for the penalty; however, this can be done via cross validation. LASSO has many desirable large sample properties including model selection consistency, which states that one can select the proper metabolites with probability tending toward the value of 1.0 if we have enough data. LASSO is known, however, to struggle in small samples with highly correlated covariates as it will simply choose one among the group of correlated variables and force the others to be zero. In these cases, related methods such as elastic net, which is a compromise between the LASSO and ridge regression procedures, can be utilized as well. These methods should provide the user with a set of metabolites that are associated with the outcome conditional on the other metabolites. Again, this may differ substantially from the variables identified by univariate techniques that do not look at conditional associations. Further, confounding factors can easily be incorporated into these high-dimensional models by including them as predictors and ensuring that their parameters are not penalized or shrunk to zero. LASSO and its variants can be implemented in the glmnet package in R [51].

Random forests is a non-parametric ensemble method that prioritizes prediction by attempting to find non-linear patterns in metabolites that can explain variation in a given outcome [52]. Random forests are very powerful tools if the relationships between the metabolites and the outcome are complex and non-linear, and has been used for both missing data imputation and outcome analysis in metabolomics [11,53]. One drawback of this approach is that, similar to PCA, it does not provide a measure of statistical significance or provide any p-value or equivalent quantity. Metabolite importance, however, can be assessed by removing metabolites one at a time, re-running the procedure, and seeing how much predictive capability was lost. This provides the analyst with a list ranking the most important metabolites but does not provide a cutoff for which metabolites are significant. Random forests can be implemented in either the randomForest or H_2_O packages in R [54,55].

Additionally, there is a vast array of methods in the machine learning literature that provide very flexible models for handling data with a large number of covariates. These approaches are appreciably powerful tools for predicting a given outcome of interest although, in many cases, at the expense of the interpretability of the resulting models. Examples of commonly used approaches include support vector machines (SVM), neural networks, and the previously mentioned random forests [56,57]. All of these methods are subject to many of the same limitations, as discussed for random forests, in that they can provide a variable importance measure but do not provide a set of variables that can be thought of as statistically significant.

A note regarding sparsity in statistical methods is warranted. A general problem of many approaches that may be used to analyze metabolomics data is that they do not easily result in a final list of “top hit” metabolites associated with a given outcome of interest. Instead, they are useful for providing heuristic measures that indicate variable importance and, in doing so, do not eliminate clinical or other covariates from the model of total covariates included in analyses. For this reason, we recommend focusing on sparse alternatives to statistical methods because, as discussed above, they are intended to directly address this issue. In this context, sparsity is based on the assumption that the number of true positives is limited, such that the contribution to variation in an outcome can be defined by a set number of non-zero values for a set number of coefficients, with the remaining being zero values. One of the most naive ways to achieve a sparse result, for instance, would be a stepwise (e.g., forward) selection, but alternate methods are preferred when the number of metabolites is far in excess to the number of observations.

## 4. Metabolomics Statistical Analysis Workflow

In addition to considering which statistical methods to apply in relating curated metabolomics data to clinical outcomes, a series of study design and data management steps are required as part of a complete analytical workflow. While not intended to be comprehensive, an overview of these steps is provided below. All examples of data analysis provided are derived from real clinical or epidemiologic cohort data unless otherwise specified.

### 4.1. Study Design

Prior to beginning any experiment, all aspects of study design should be carefully considered. This process includes many considerations. First, the choice between univariate vs. multivariate methods is largely driven by the scientific question of interest. If the primary objective of the study is to identify all metabolites associated with a particular outcome, the univariate methods preferable. If the primary objective instead is to utilize potentially multiple metabolites to predict the outcome of interest, then multivariate methods will be preferred. Next, a decision on whether metabolite predictors of a given outcome will be investigated in a case-control (and, if so, the number of cases and controls), a case-cohort, or total cohort design. In this step, it is critically important to consider the number of outcome occurrences (i.e., cases) available for analysis and the anticipated effect size for metabolomic variation in relation to case status. For example, for a rare outcome (i.e., 1% prevalence in the study population), there is greater statistical power to detect a given effect size via a case-control study design of *n* = 42 (21 cases vs. 21 controls) than via an unselected sample of *n* = 1000 (10 cases vs. 990 controls). Also, it is necessary to decide on whether the metabolomics profiling method to be used will be nontargeted or targeted and, if targeted, the type of targeted approach. This decision will impact the approximate number of metabolites that will be measured. Taken together, information on the total number of observations, frequency or distribution of outcomes, and number of metabolites measured will determine the extent to which the experiment will be adequately powered for detecting clinically significant associations of interest. As an example, when using Bonferroni-like approaches to control type-I error, the difference between testing 200 vs. 2000 metabolites results means comparing the results of statistical tests against *p*-values of 0.00,025 (=0.05/200) vs. 0.000,025 (=0.05/2000). A study designed to detect a given magnitude of association with conventional 80% power for the former would have only 60% power in the latter scenario due to the more stringent threshold for declaring statistical significance. As a result, the magnitude of the minimal association that the latter study would be able to detect would be approximately 15% greater than originally planned and the sample size would need to be increased by 30% in order to regain sufficient power to detect the originally intended magnitude of association. Similar considerations are needed for the analysis of continuous as opposed to binary outcomes. For a normally distributed outcome, the sample size required to detect a difference between groups will be at least 50% larger if the outcome is first dichotomized and analyzed as binary rather than continuous. Overall, this step is essential for conducting analysis of metabolomics data wherein the number of metabolites measurable typically far exceeds the number of individuals studied within a given experiment.

### 4.2. Data Management

It is becoming increasingly recognized that several key data management steps are extremely important for ensuring not only the integrity but also feasibility of metabolomics data analysis. For instance, it is always important to perform a careful assessment for batch-to-batch variability that can often persist even after preprocessing steps have been successfully completed (e.g., for alignment of mass spectral features) [58]. While an area of ongoing research, informed decisions can be made regarding whether or not to normalize a given dataset to internal standards, to pooled plasma measures, to both, or to neither [59,60,61].

**Figure 1:**
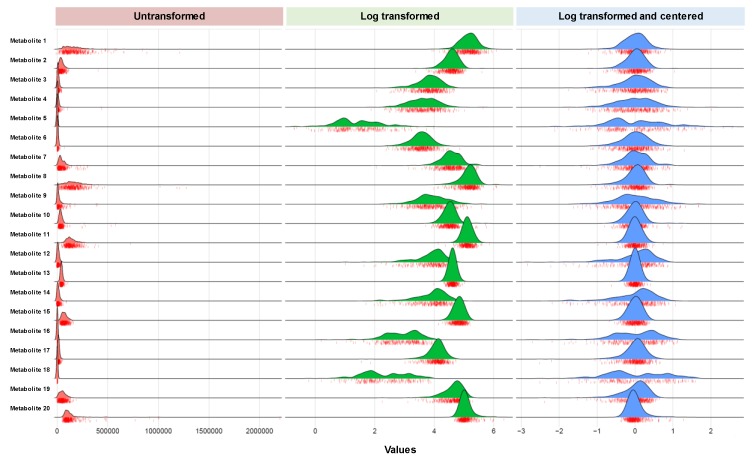
Metabolite data transformation and centering. A frequently used approach for managing metabolite data collected in a large human cohort study involves log transforming each metabolite measures and centering the data on plate median to account for batch to batch variation. Interestingly, variable transformation can reveal multi-modal distributions.

All datasets should be examined for their data structure, including the distribution and type of missingness across metabolites and across individuals. Different approaches to handling missingness may be suitable depending on the types of metabolites profiled (e.g., known to be rare, low abundant, or technically difficult to detect). As discussed in Section 2, imputation strategies are generally preferred to more ad hoc approaches such as simply filling in missing values as zero or half the minimum observed values. Subset analyses are always possible, and some investigators have elected to exclude analytes with substantial missingness from all analyses. All approaches to handling missing values may introduce bias, depending on the method and cohort characteristics. While we have provided suggestions in Section 2, we also feel that sensitivity analysis is crucial to ensure that the results are robust to the chosen method for addressing missingness. Researchers should look at their primary research question and evaluate whether their conclusions change substantially across differing methods for missing data. After having addressed issues related to missingness, metabolite variables typically benefit from applying transformation and scaling to allow for appropriateness and comparability of statistical analyses (Figure 1). Although natural log transformation is commonly used for most or all metabolite variables in a given dataset, given the typical high proportion of metabolites with right-skewed distribution, this approach may not be optimal for all variables [14]. We recommend performing a natural log transformation of all metabolite variables and then assessing the skewness before and after transformation. For those metabolites for which the skewness is not improved after transformation, we recommend retaining the original untransformed variables for further analysis, with transformed values used for the other metabolites. While standardization of transformed variables is also commonly used, so that magnitudes of effect are comparable across models, other approaches such as Pareto or level scaling may be more appropriate for certain study designs (i.e., taking into consideration the main research questions and outcomes of interest) [14].

### 4.3. Optional Simulation Analyses

**Figure 2:**
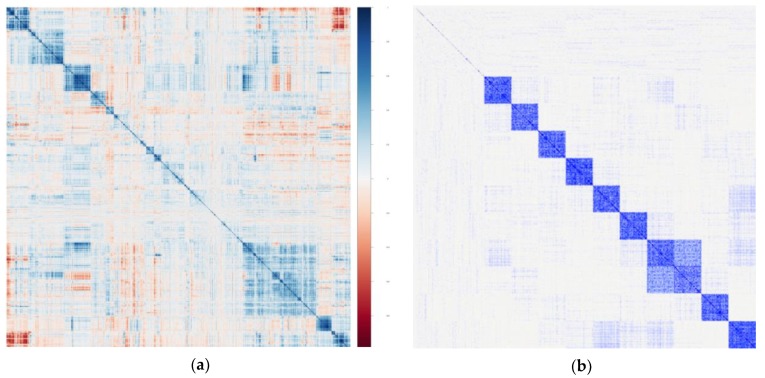
Actual and simulated metabolomics data. Previously analyzed data, or prior detailed knowledge of the structure of metabolomics data collected from an existing large epidemiologic cohort study (**a**) can be used to construct simulated data that mimics the data structure observed from real measures (**b**). These simulated data can be used to estimate statistical power, based on one or more methods of analyses, for planning the design of a future study.

If feasible, an ideal approach is to develop a dataset that simulates the design, size, and features of the study at hand, including the number of metabolites measured and the expected or known number of outcomes. Simulated data allow for comparisons of performance of the different statistical methods, so that the most optimal method can be selected for a given study. Simulations are especially useful for guiding the design of studies that are in the early planning phases, particularly those for which the number of metabolites and the number of study subjects are yet to be determined. To perform such simulations in a relevant manner, some data structural features of the data must be known. For instance, if prior data are already available, the empirical distribution of the metabolites can be used (Figure 2). One can resample the rows of the true data to obtain simulated data that closely replicate the true data, and then an outcome can be generated assuming a pre-specified relationship with the metabolites. Another possibility is to learn the covariance matrix among all the metabolites in the study and then draw values from a multivariate normal distribution with this covariance to create simulated metabolite variables. If an example of true data is not available and simulations are being used to design a new study, then a dataset from previously conducted related study may be used to guide the simulation design. With simulated datasets in hand, it is possible to obtain power calculations that are relevant and applicable for planning study design. One can simulate metabolites and an outcome and repeat this process many times over while recording the percentage of times a metabolite marker of interest is identified, which provides the power at the given sample size. This process can be performed iteratively using different sample sizes, and different pre-specified associations between the metabolites and outcome, to gain a better understanding of the power available to identify signals in the planned study.

### 4.4. Cross-Sectional or Prospective Analyses (i.e., Outcomes Analyses)

After selecting the most appropriate statistical approach, based on prior experience or the results of simulation analyses (Section 4.3 above), applying a method that includes internal cross-validation should be considered. Cross-validation procedures are intended to optimize generalizability and reduce variability of results by performing repeated analyses on different partitions of the dataset and then averaging the results to estimate a final model. For instance, in k-fold cross-validation, the total dataset is randomly divided into k equally sized subsets, and k-1 subsets are analyzed while reserving a different single subset for validation during each iteration. An alternative to cross-validation is conventional validation or dividing the original dataset into training and validation subsets (e.g., 2/3, 1/3) while assuming the original dataset size is large enough to accommodate this approach and a separate validation cohort is not available. Yet another option for assessing generalizability is to apply other statistical methods and compare results [62], as an investigator can feel more confident about results that are consistently obtained irrespective of the statistical technique used (see Section 4.4 below). It should be noted that this only ensures the robustness to the choice of statistical approach, but does not guarantee accuracy of the results. There are a number of issues in the collection and pre-processing of data that could lead to systematic errors, and no statistical approach can correct for these. In these cases, the statistical approaches might all lead to the same, but incorrect, answers. For this reason, it is crucial to carefully design the study and assess the robustness of all stages of the workflow.

### 4.5. Real-Life Application of Multiple Statistical Methods

**Figure 3:**
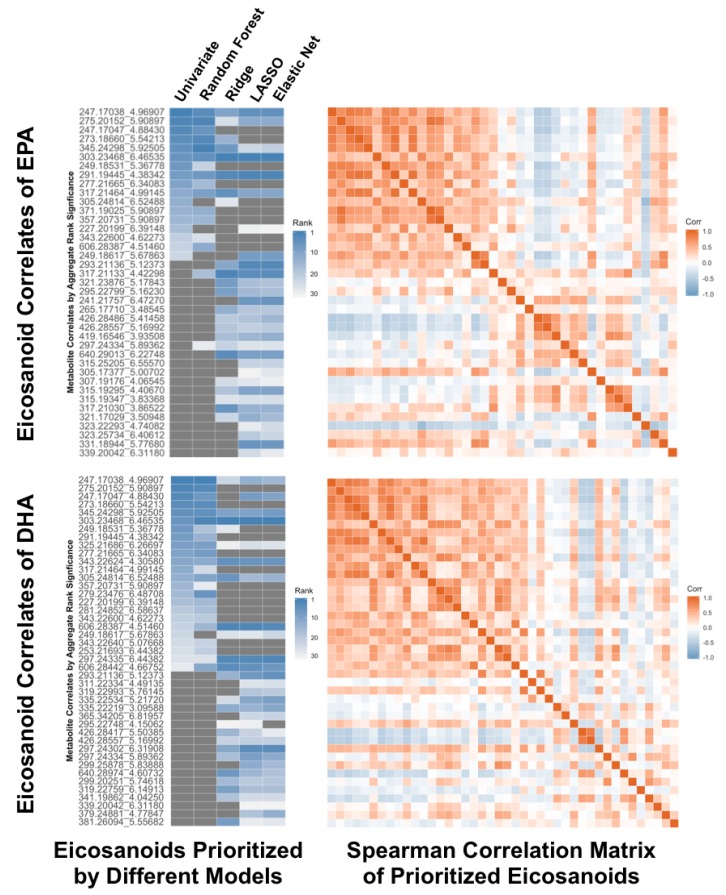
Using multiple statistical methods to evaluate results in a real-life application involving analyses of large cohort metabolite data. We related a panel of bioactive lipid molecule metabolites (i.e., eicosanoids) to putative derivative substrates (i.e., eicosapentaenoic acid (EPA) and docosahexaenoic acid (DHA)), which were considered in all analyses as the outcomes of clinical relevance and interest. We used multiple different statistical methods and compared results. Metabolites are denoted by mass-to-charge (*m*/*z*) ratio and retention time (rt, in minutes) using the *m*/*z*_rt convention, and are listed in rank order for each outcome (EPA or DHA) according to performance metrics provided by each model.

Herein, we provide an example of how data analysis results can vary when different statistical modeling approaches are applied to real-life metabolomics data. Specifically, we studied *N* = 1497 adult participants of the Framingham Heart Study Offspring cohort (age 67 ± 10 years, 55% women) who had fasting plasma collected at their eighth examination [63]. We used a direct nontargeted mass spectrometry profiling method [64] to quantify *P* = 547 metabolites of the eicosanoid family (a subset of bioactive lipids) in the collected plasma samples. These metabolites included eicosanoid species that are known to be predominantly derived from eicosapentaenoic acid (EPA) (20:5, n-3) and docosahexaenoic acid (DHA) (22:6, n-3), each of which hs been linked to a variety of clinically important cardiometabolic traits [65]. The extent to which specific eicosanoids (as potential derivatives) are more or less related to EPA versus DHA (as potential initial substrate molecules) has not be clarified, and so we used different statistical modeling approaches to identify and prioritize eicosanoids of interest. As shown in Figure 3, different modeling methods produced distinct results. We observed relative consensus in results between the univariate approaches and the random forest approach, and among the multivariate approaches, but less so across these groups of methods. Intrestingly, univariate and random forest approaches tended to select metabolites within one intercorrelated group, and multivariate approaches tended to select metabolites across multiple clusters of intercorrelated metabolites (positive correlations shown in red; negative correlations shown in blue). Notably, the top metabolites from the univariate approaches are all highly correlated, suggesting that multivariate approaches are selecting metabolites after conditioning on the other predictors, which likely contributes to the observed variability in results. Additionally, discordant results likely reflect different assumptions and features between methods, such as the assumption of linearity of association between a predictor and outcome for conventional regression models, which random forests does not rely on. These findings highlight the potential for multivariate methods, by contrast, to select putative orthogonal rather than common pathways of biological interest. In this particular example, the distinction is relevant for identifying potential eicosanoid pathways that may be enriched versus diversified with respect to their reliance on either EPA or DHA metabolism and their downstream effects—which are likely to be similar to each other in some respects and different in others [65].

### 4.6. Prioritization of Results for Follow-Up Investigations

When using a nontargeted MS method, the majority of significantly associated metabolites will invariably be novel (i.e., previously unidentified molecular species). Thus, an imperative next step in the scientific process is to identify such novel molecules of potential clinical importance. However, the process of identifying the specific chemical structure of a previously unknown small molecule is time-consuming and potentially very resource-intensive, depending on the molecule’s relative abundance in available biospecimens and other characteristic features. Therefore, the results of statistical analyses should ideally be robust and convincing before they are used to direct efforts made towards novel small molecule identification. To this end, several statistical approaches to prioritizing small molecules for follow-up identification are possible. Univariate approaches and p-values provide a simple and effective tool for prioritizing metabolites that are associated with an outcome. We have discussed how other approaches based on high-dimensional models can lead to another set of important metabolites that are important for predicting the outcome when conditioning on other metabolite values. These multivariate approaches can be quite useful when the metabolites are highly correlated, though may only select one out of a group of highly correlated variables associated with an outcome. Understanding the differences between these prioritized variables is crucial and the choice of which to use depends on the research question of interest. Beyond cross-validation or conventional validation within a dataset, external replication in a separate cohort is ideal, and even further value can be gained from using different metabolomics platforms [8]. In addition, confirmatory results from performing different types of statistical analyses (e.g., traditional and non-traditional) in both training and validation cohorts may be informative.

## 5. Conclusions

High-throughput metabolomics data provide an exciting area of research for scientific discovery, but they are accompanied by a number of statistical challenges that must be properly addressed to infer meaningful results from such complex data. Herein, we have reviewed and outlined practical solutions for many of the common problems found in metabolomics data analysis. While outlining some guidelines for future researchers on how to address these issues, the optimal solution at any given stage of data management and analysis depends on the size and design of the specific data and study at hand, as well as the research questions being addressed. Thus, a critical aspect of any analysis is explicit recognition of the strengths and weaknesses of the selected approaches as well as a complete understanding of the assumptions that are implicit for every decision made at each step of the analysis workflow. Many important decisions, such as those related to handling of missing data and transforming skewed data, can have substantial and meaningful impacts on the final analysis results. Therefore, we recommend that researchers perform sensitivity analyses with respect to such decisions to assess the robustness of their results in the context of such subjective choices. In addition, any findings from primary analyses should be replicated in additional studies to confirm their potential to serve as meaningful scientific findings, irrespective of the statistical decisions made.

While we have reviewed potential solutions for the problems that are often encountered in human metabolomics studies, many of these issues do not have definitive answers, and this presents many possibilities for future methods research that can aim to improve decision making at each stage of the analysis workflow. These are all open areas of research, and finding optimal solutions will lead to substantial improvements in analysis, reproducibility, and overall contributions in the field of human metabolomics investigation.

## Figures and Tables

**Table 1:** Statistical considerations for human metabolomics data.

Consideration	Notes and Examples
Missingness	Patterns of missing values tend to be non-random and are even sometimes predictable. For example, missing values may often but not always be more frequent for metabolites that are intrinsically low in abundance when measured from a given tissue type.
	Missingness may be due to biological and/or technical reasons.
Data distributions	Many but not all metabolites tend to demonstrate right-skewed distributions in most types of human studies (e.g., healthy controls or disease-specific referral samples).
	Certain metabolites will display a substantial proportion of zero values that may be considered true zero values based on biology (an issue to be considered along with but distinguished from missingness).
Intercorrelations	Intercorrelations between metabolites may well reflect clustering of small molecules by known or (mostly) unknown biological pathways.
	Intercorrelations will vary widely depending on a given exposure or background, chronic disease status, and other yet unidentified factors.
	Intercorrelations will also vary depending on the underlying mass spectrometry (MS) method used to create a given dataset (i.e., nontargeted vs. targeted, and the specific technical methods used).
Time-dependence	Whereas a portion of the human metabolome changes dynamically in response to acute perturbation or stress, many other metabolites display variation only over several days to weeks in response to subacute perturbations; other portions of the metabolome may yet exhibit relatively little change over time, except in response to major chronic exposures.
Confounding factors	Metabolite values will vary in response to factors that are measurable as well as factors that are not easily measurable for a given study, such as acute and chronic dietary patterns, microbiota, and environmental exposures.

**Table 2:** Statistical analysis methods for outcomes analyses of human metabolomics data.

Method	Univariate or Multivariate	Handling Binary Outcome	Handling Continuous Outcome	*p* Value for Significant Metabolites	Metabolite Selection	Advantages	Disadvantages
Multiple tests (e.g., univariate linear regression) with Bonferroni correction	Univariate	Yes	Yes	Yes	Yes	Simple, easy to use and interpret results	Very conservative and does not account for intercorrelation
Multiple tests with false discovery rate (FDR)	Univariate	Yes	Yes	Yes	Yes	Simple, easy to use, less conservative than Bonferroni correction	Does not account for intercorrelation among features
Principal component analysis (PCA)	Multivariate	Yes	Yes	No	No	Effective for variable reduction	No intrinsic clarity on how to select or rank variables
Sparse partial least squares (SPLS)	Multivariate	No	Yes	No	Yes	Can quickly find a subset of variables that predicts the outcome well	Multiple tuning parameters are needed to be chosen via cross validation
Linear discriminant analysis (LDA)	Multivariate	Yes	No	No	Yes	Simple and works for categorical outcomes	Can not handle large numbers of features
Least absolute shrinkage and selection operator (LASSO)	Multivariate	Yes	Yes	No	Yes	Can quickly find a subset of variables that predicts the outcome well	May not perform well for metabolite selection when the features are highly correlated
Random forests and other machine learning approaches	Multivariate	Yes	Yes	No	No	Can find complex relationships between variables	If data is truly linear, this will be less efficient
